# Delivery of Community Support Services for Older Adults in Social Housing

**DOI:** 10.1093/geroni/igaa057.3348

**Published:** 2020-12-16

**Authors:** Matthew Yau, Christine Sheppard, Jocelyn Charles, Andrea Austen, Sander Hitzig

**Affiliations:** 1 University of Toronto, University of Toronto, Ontario, Canada; 2 Sunnybrook Research Institute, Toronto, Ontario, Canada; 3 Sunnybrook Health Sciences, Toronto, Canada; 4 City of Toronto, Toronto, Ontario, Canada; 5 Sunnybrook Hospital, North York, Ontario, Canada

## Abstract

Community support services are an integral component of aging in place. In social housing, older adult tenants struggle to access these services due to the siloed nature of housing and health services. This study aims to describe the relationship between community support services and social housing for older adults and examine ways to optimize delivery. Data on government-funded community support services delivered to 74 seniors’ social housing buildings in Toronto, Ontario was analyzed. Neighbourhood profile data for each building was also collected, and correlational analyses were used to examine the link between neighbourhood characteristics and service delivery. Fifty-six community agencies provided 5,976 units of services across 17 service categories, most commonly mental health supports, case management and congregate dining. On average, each building was supported by nine agencies that provided 80 units of service across 10 service categories. Buildings in neighbourhoods with a higher proportion of low-income older adults had more agencies providing on-site services (r = .275, p < .05), while those in neighbourhoods with more immigrants (r = -.417, p < .01), non-English speakers (r = -.325, p < .01), and visible minorities (r = -.381, p < .01) received fewer services. Findings point to a lack of coordination between service providers, with multiple agencies offering duplicative services within the same building. Vulnerable seniors from equity-seeking groups, including those who do not speak English and recent immigrants, may be excluded from many services, and future service delivery for seniors should strive to address disparities in availability and access.

